# Alteration of Platelet Count in Patients with Severe Non-*Plasmodium falciparum* Malaria: A Systematic Review and Meta-Analysis

**DOI:** 10.3390/biology10121275

**Published:** 2021-12-05

**Authors:** Aongart Mahittikorn, Frederick Ramirez Masangkay, Kwuntida Uthaisar Kotepui, Wanida Mala, Giovanni De Jesus Milanez, Polrat Wilairatana, Manas Kotepui

**Affiliations:** 1 Department of Protozoology, Faculty of Tropical Medicine, Mahidol University, Bangkok 10400, Thailand; aongart.mah@mahidol.ac.th; 2 Department of Medical Technology, Institute of Arts and Sciences, Far Eastern University-Manila, Manila 1008, Philippines; frederick_masangkay2002@yahoo.com; 3 Medical Technology, School of Allied Health Sciences, Walailak University, Tha Sala, Nakhon Si Thammarat 80160, Thailand; kwuntida.ut@wu.ac.th (K.U.K.); wanida.ma@wu.ac.th (W.M.); 4 Department of Medical Technology, Faculty of Pharmacy, University of Santo Tomas, Manila 1008, Philippines; Gmilanez81@gmail.com; 5 Department of Clinical Tropical Medicine, Faculty of Tropical Medicine, Mahidol University, Bangkok 10400, Thailand; polrat.wil@mahidol.ac.th

**Keywords:** platelet, thrombocytopenia, falciparum, malaria

## Abstract

**Simple Summary:**

Alteration of platelet count is frequently seen in patients with malaria. However, the platelet biology under severe pathological conditions like severe malaria remains unclear. The present systematic review collated literatures and synthesized the evidence regarding the risks of severe and profound thrombocytopenia in patients with severe non-*Plasmodium falciparum* malaria. We found that the high prevalence of severe and profound thrombocytopenia in patients with severe non-*P. falciparum* malaria at 47% and 20%, respectively. Moreover, deaths were seen in patients with severe and profound thrombocytopenia at 11%. Therefore, the present systematic review provided the new insight into the clinical relevance of severe and profound thrombocytopenia in diagnosing and managing non-*P. falciparum* malaria. Severe thrombocytopenia should serve as a warning sign of increased chances of complications. Meanwhile, profound thrombocytopenia should serve as a marker of mortality among patients with non-*P. falciparum* malaria.

**Abstract:**

The understanding of platelet biology under physiological and pathological conditions like malaria infection is critical importance in the context of the disease outcome or model systems used. The importance of severe thrombocytopenia (platelet count < 50,000 cells (µL) and profound thrombocytopenia (platelet count < 20,000 cells/µL) in malaria patients remains unclear. This study aimed to synthesize evidence regarding the risks of severe and profound thrombocytopenia in patients with severe non-*Plasmodium falciparum* malaria. Our overall aim was to identify potential indicators of severe non-*P. falciparum* malaria and the *Plasmodium* species that cause severe outcomes. This systematic review was registered at the International Prospective Register of Systematic Reviews (PROSPERO) under registration ID CRD42020196541. Studies were identified from previous systematic reviews (*n* = 5) and the MEDLINE, Scopus, and Web of Science databases from 9 June 2019 to 9 June 2020. Studies were included if they reported the outcome of severe non-Plasmodium species infection, as defined by the World Health Organization (WHO) criteria, in patients with known platelet counts and/or severe and profound thrombocytopenia. The risk of bias was assessed using the Newcastle–Ottawa Scale (NOS). Data were pooled, and pooled prevalence (PP) and pooled odds ratios (ORs) were calculated using random effects models. Of the 118 studies identified from previous meta-nalyses, 21 met the inclusion criteria. Of the 4807 studies identified from the databases, three met the inclusion criteria. Nine studies identified from reference lists and other sources also met the inclusion criteria. The results of 33 studies reporting the outcomes of patients with severe *P. vivax* and *P. knowlesi* infection were pooled for meta-analysis. The PP of severe thrombocytopenia (reported in 21 studies) was estimated at 47% (95% confidence interval (CI): 33–61%, I^2^: 96.5%), while that of profound thrombocytopenia (reported in 13 studies) was estimated at 20% (95% CI: 14–27%, 85.2%). The pooled weighted mean difference (WMD) in platelet counts between severe uncomplicated *Plasmodium* infections (reported in 11 studies) was estimated at −28.51% (95% CI: −40.35–61%, I^2^: 97.7%), while the pooled WMD in platelet counts between severe non-*Plasmodium* and severe *P. falciparum* infections (reported in eight studies) was estimated at −3.83% (95% CI: −13.90–6.25%, I^2^: 85.2%). The pooled OR for severe/profound thrombocytopenia comparing severe to uncomplicated *Plasmodium* infection was 2.92 (95% CI: 2.24–3.81, I^2^: 39.9%). The PP of death from severe and profound thrombocytopenia was estimated at 11% (95% CI: 0–22%). These results suggest that individuals with severe non-*P. falciparum* infection (particularly *P. vivax* and *P. knowlesi*) who exhibit severe or profound thrombocytopenia should be regarded as high risk, and should be treated for severe malaria according to current WHO guidelines. In addition, severe or profound thrombocytopenia coupled with other clinical and microscopic parameters can significantly improve malaria diagnosis, enhance the timely treatment of malaria infections, and reduce the morbidity and mortality of severe non-*P. falciparum* malaria.

## 1. Introduction

An understanding of platelet biology under physiological and pathological conditions like malaria infection is of critical importance in the context of the disease outcome or model systems used. Platelets are produced from megakaryocytes and play a crucial role in hemostasis and thrombus formation [1]. Thrombocytopenia (platelet count < 100,000 cells/µL) is common in patients with malaria [2,3,4]. In patients with *Plasmodium falciparum* and *Plasmodium* vivax infections, thrombocytopenia is associated with increased mortality [5,6]. The proposed pathogenic mechanisms during *P*. *falciparum* malaria are increased platelet activation and consumption by factors such as the cytoadherence of infected erythrocytes and subsequent endothelial damage [7], platelet accumulation in the microvasculature [8], bone marrow alterations, and antibody-mediated platelet destruction [9]. Severe thrombocytopenia is defined as a platelet count of <50,000 cells/µL, while profound thrombocytopenia is defined as a platelet count of <20,000 cells/µL [10]. Severe or profound thrombocytopenia has been reported in patients with *P*. *falciparum* and *P*. *vivax* malaria and is associated with bleeding [11] and disseminated intravascular coagulation [12].

Severe malaria is defined as the presence of *Plasmodium* parasitemia and one or more of the following manifestations: impaired consciousness, prostration, acidosis, hypoglycemia, severe malarial anemia, renal impairment, jaundice, pulmonary edema, significant bleeding, shock, or hyperparasitemia [13]. Severe or profound thrombocytopenia has been widely documented in patients with severe *P*. *falciparum* malaria, although it is not included in the current World Health Organization (WHO) criteria for severe *P*. *falciparum* malaria [13]. A previous study reported that adults with *P*. *falciparum* malaria and profound thrombocytopenia were five times more likely to die than patients with higher platelet counts [14]. Although severe thrombocytopenia has been widely recognized as a feature of patients with severe *P*. *falciparum* infection, information on the prevalence of severe or profound thrombocytopenia in patients with *non*-*P*. *falciparum* malaria (including *P*. *vivax, P*. *malariae, P*. *ovale,* and *P*. *knowlesi* infection) is limited. The most recent systematic review on severe thrombocytopenia in *P*. *vivax* malaria demonstrated that severe thrombocytopenia was equally common in *P*. *vivax* and *P*. *falciparum* malaria [15]. However, the results were interpreted based on a small number of studies with low sample sizes, and the estimates had wide confidence intervals. In addition, the study did not report the outcomes of severe *P*. *vivax* infection. To clarify this issue, we conducted a meta-analysis of studies reporting severe or profound thrombocytopenia in patients with severe *P*. *vivax*, *P*. *malariae*, *P*. *ovale*, and *P*. *knowlesi* malaria. Our goal was to assess whether severe or profound thrombocytopenia could inform clinical decision-making, and whether the degree of thrombocytopenia had predictive utility independent of current clinical and laboratory prognostic indices.

## 2. Methods

### 2.1. Study Registration

This systematic review was registered at the International Prospective Register of Systematic Reviews (PROSPERO) under registration ID CRD42020196541.

### 2.2. Types of Studies and Inclusion and Exclusion Criteria

Original studies were included if they were observational, prospective, or retrospective in design, and reported outcomes of patients with: (1) severe malaria as defined by the WHO (impaired consciousness, jaundice, pulmonary edema, acute renal failure, severe anemia, bleeding, acidosis, hyperparasitemia, shock, respiratory distress, or hypoglycemia); (2) non-*Plasmodium falciparum* including *P*. *vivax*, *P*. *malariae*, *P*. *ovale*, and *P*. *knowlesi* infections; (3) severe thrombocytopenia (platelet count < 50,000 cells/µL) or profound thrombocytopenia (platelet count < 20,000 cells/µL); (4) severe and uncomplicated malaria with known platelet counts; or (5) severe *P*. *falciparum* and non-*P*. *falciparum* infections with known platelet counts. The following types of studies were excluded: (1) studies examining severe non-*Plasmodium* infection co-morbidity with other diseases, (2) studies conducted among pregnant patients, and (3) studies in which platelet counts could not be extracted. The systematic review was performed following the Preferred Reporting Items for Systematic Reviews and Meta-Analyses (PRISMA) guidelines (see PRISMA Checklist S1).

### 2.3. Search Strategy and Data Extraction

Potentially relevant studies were identified from three sources. The first source of studies was previous systematic reviews (*n* = 5) reporting outcomes of severe *P*. *vivax*, *P*. *malariae*, *P*. *ovale*, and *P*. *knowlesi* infections. The second source of studies was a systematic search of relevant databases (MEDLINE, Scopus, and ISI Web of Science) between 1 June 2020 and 9 June 2020 using MeSH headings (Severe OR complicated OR complication) AND (malaria OR plasmodium) AND (thrombocytopenia OR “low platelet”), with no restrictions on language. The third source of studies was the reference lists of the included studies as well as other sources (e.g., Google Scholar). Two authors (AM and MK) independently screened the titles and abstracts. Any disagreements in full-text article selection were resolved by consensus. Potentially relevant full-text articles were obtained, read, and extracted by two independent authors concerning the inclusion criteria. Data were extracted into a standardized form using an Excel spreadsheet. For all studies, data on authors, year of publication, study location, year of publication, and study design were extracted. In addition, data on mean/median age of participants, male percentage, mean/median platelet count, parasitemia level, number of patients with severe and profound thrombocytopenia, *Plasmodium* species, number of patients with severe malaria, number of patients with severe or profound thrombocytopenia, and number of deaths were extracted. Data extraction was undertaken by one author and checked by all authors.

### 2.4. Assessment of Risk of Bias

The risk of bias was independently assessed by two authors (A.M. and M.K.). As the included studies were observational retrospective, prospective, and case-control studies, the Newcastle–Ottawa Scale (NOS) tool was used [16]. The NOS tool allows for quality assessment in three domains, including the selection of study groups, comparability of groups, and ascertainment of the outcome. The NOS used as the primary outcome in this meta-analysis was the pooled prevalence (PP) of severe or profound thrombocytopenia among one group (patients with severe non-*P*. *falciparum* malaria) and not the comparator groups. Therefore, only three domains were used to assess the included studies: case definition, representativeness of cases, and ascertainment of the outcome. The risk of bias was reported as low (1 star), moderate (2 stars), or high (3 stars) based on the total of the three scores retrieved from each domain. Any discrepancies in ratings were addressed in the discussion.

### 2.5. Data Synthesis and Analysis

The following three outcomes were analyzed by meta-analysis: (1) PP and 95% confidence interval (CI) of severe and profound thrombocytopenia in individuals with severe non-*P*. *falciparum* infection, estimated using the random-effects DerSimonian and Laird models [17]; (2) pooled odds ratios (ORs) in individuals with severe non-*P*. *falciparum* infection compared with individuals with uncomplicated non-*P*. *falciparum* infection, estimated using fixed- or random-effects models depending on the level of heterogeneity; (3) pooled ORs in individuals with severe non-*P*. *falciparum* infection compared with individuals with severe *P*. *falciparum* infection, estimated using fixed- or random-effects models depending on the level of heterogeneity. The heterogeneity and level of heterogeneity of studies were assessed using Cochran’s Q and I^2^ (inconsistency) statistics, respectively. Publication bias was evaluated using visual inspection of funnel plots and Egger’s tests. If any small-study effects were observed, a further contour-enhanced funnel plot was generated to assess if asymmetry resulted from publication bias or other factors. The meta-analysis was carried out using Stata software. To explore whether a single study affected the heterogeneity or the conclusions of the meta-analysis, sensitivity analyses omitting each study were performed in turn. Subgroup analyses were conducted to evaluate potential sources of heterogeneity.

## 3. Results

**Figure 1:**
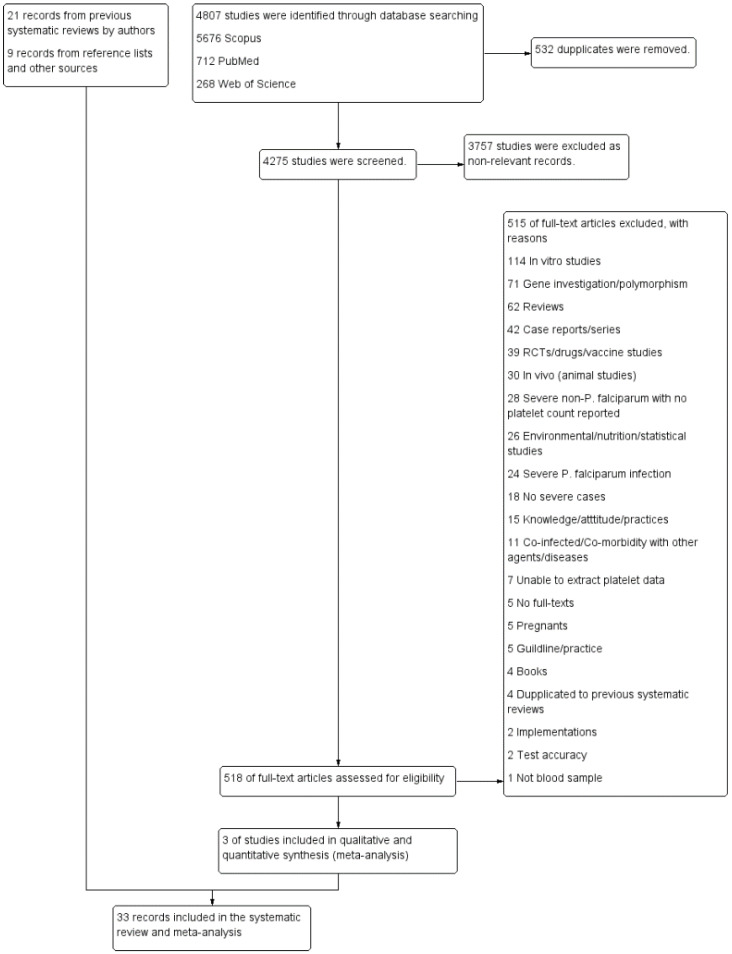
Study flow diagram. The diagram showed the selection process of potentially relevant studies. When duplicates (532 studies) were removed, the remaining studies (4275 studies) were screened for titles and abstracts, and 3757 non-relevant studies were excluded. Next, 518 studies were examined in full-text, and then 515 studies were excluded for specific reasons. Three studies met the eligibility criteria, and 33 studies retrieved from other sources were included in the meta-analysis.

Twenty-one studies reporting the outcomes of severe non-*P*. *falciparum* malaria were retrieved from previous systematic reviews and meta-analyses [18,19,20,21,22]. As previous systematic reviews included studies dating until 2019, additional searches between 2019 and 2020 were also performed, which identified 4807 unique articles. Of the titles and abstracts screened, 518 articles were selected for full-text screening (Figure 1). Three studies were eligible for inclusion. Additional searches of reference lists and other sources identified nine articles meeting the inclusion criteria. A total of 33 studies were included in this systematic review and assessed for methodological quality [10,23,24,25,26,27,28,29,30,31,32,33,34,35,36,37,38,39,40,41,42,43,44,45,46,47,48,49,50,51,52,53,54].

### 3.1. Characteristics of the Included Studies

Thirty-three studies from Asia (India [10,23,25,31,35,36,37,38,39,40,43,46,47,49,50,51], Malaysia [29,30,53,54], Indonesia [32], Papua New Guinea [42], and Republic of Korea [45]) and South America (Brazil [24,33,34,41,48,52] and Colombia [26,27,28,44]) reported data on 1495 individuals with severe non-*P*. *falciparum* malaria. The included studies were prospective or retrospective observational studies, case-control studies, and cohort studies. All studies excluded individuals with *P*. *falciparum* single or mixed infections. Most of the included studies reported the outcomes of severe non-*P*. *falciparum* malaria in adults, while Gupta et al., 2016 [36], Lanca et al., 2012 [41], and Singh et al., 2011 [50] reported outcomes of severe non-*P*. *falciparum* malaria in children younger than 7 years. Most of the included studies reported outcomes of predominantly male individuals who developed severe non-*P*. *falciparum* malaria, while Sharma et al., 2012 and Singh et al., 2011 reported a higher proportion of women who developed severe non-*P*. *falciparum* malaria. All non-*P*. *falciparum* malaria cases reported in the included studies involved *P*. *vivax* and *P. knowlesi* malaria. Of the included studies, 30 [10,23,24,25,26,27,28,30,31,32,33,34,35,36,37,38,39,40,41,42,43,44,45,46,47,48,49,50,51,52] reported outcomes of 1371 cases of severe *P*. *vivax* single infections, while four [29,30,53,54] reported outcomes of 124 cases with severe *P*. *knowlesi* infections. Barber et al., 2013 [30] reported outcomes of patients with both severe *P*. *vivax* and severe *P*. *knowlesi* infections. Other characteristics of the included studies are shown in Table S1.

### 3.2. Risk of Bias of Individual Studies

Of the 33 included studies that were assessed for quality, 28 [10,23,24,25,26,27,28,29,30,32,33,35,36,37,38,39,40,41,42,43,44,45,46,47,48,50,51,52] were rated as having the highest quality (3 stars) while the remaining five studies [31,33,49,53,54] were rated as moderate quality (2 stars) because of the unclear case selection criteria for inclusion (Table S2).

### 3.3. Prevalence of Severe Thrombocytopenia in Severe Non-P. falciparum Malaria

**Figure 2:**
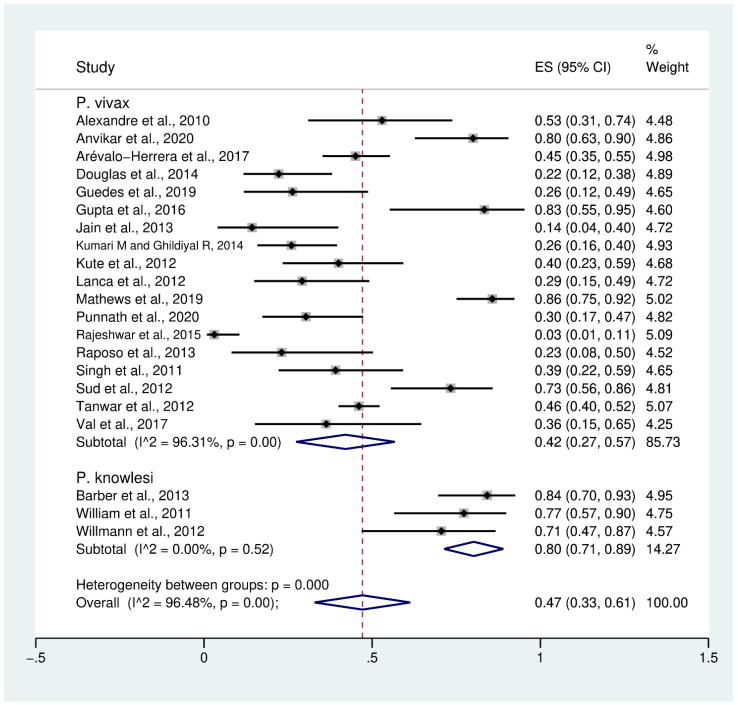
Prevalence of severe thrombocytopenia in patients with severe non-*P*. *falciparum* malaria. Abbreviations: ES, effect size; CI, confidence interval; black diamond symbol, point estimate; dashed line: pooled prevalence of severe thrombocytopenia; I^2^, level of heterogeneity; *p* = 0.00 or less than 0.05, significant heterogeneity.

Data on severe thrombocytopenia (349 cases) in patients with severe *P*. *vivax* malaria (808 cases) from 18 studies [10,24,25,28,32,34,36,37,39,40,41,43,46,47,48,50,51,52] were extracted and pooled in the meta-analysis of proportion. The PP of severe thrombocytopenia in patients with severe *P*. *vivax* malaria was 42% (95% CI: 27–57%, I^2^: 96.3%). The prevalence reported in individual studies varied highly (range: 3–86%). Data on severe thrombocytopenia in patients with severe *P*. *knowlesi* malaria were available from three studies [30,53,54]. The PP of severe thrombocytopenia (61 cases) in patients with severe *P*. *knowlesi* malaria (77 cases) was 80% (95% CI: 71–89%, I^2^: 0%). The prevalence reported in the three individual studies of severe *P*. *knowlesi* malaria was consistent, ranging from 71% to 84%. Overall, the PP of severe thrombocytopenia (440 cases) in patients with severe *P*. *vivax* and severe *P*. *knowlesi* malaria (885 cases) was 47% (95% CI: 33–61%, I^2^: 96.5%) (Figure 2).

### 3.4. Prevalence of Profound Thrombocytopenia in Severe Non-P. falciparum Malaria

**Figure 3:**
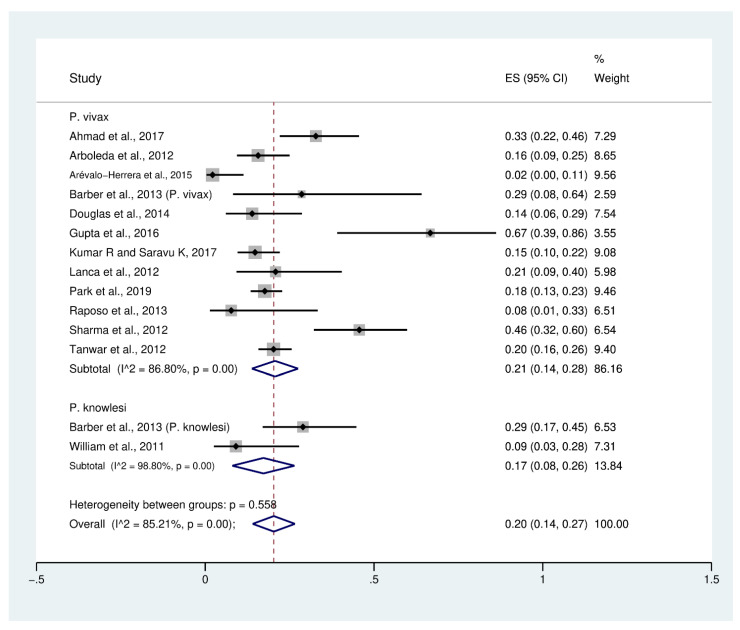
Prevalence of profound thrombocytopenia in patients with severe non-*P*. *falciparum* malaria. Abbreviations: ES, effect size; CI, confidence interval; black diamond symbol: point estimate; dashed line: pooled prevalence of severe thrombocytopenia; I^2^, level of heterogeneity; *p* = 0.00 or less than 0.05, significant heterogeneity.

Data on profound thrombocytopenia (189 cases) in patients with severe *P*. *vivax* malaria (954 cases) from 12 studies [10,23,26,27,30,32,36,38,41,45,48,49] were extracted and pooled in the meta-analysis of proportion. The PP of profound thrombocytopenia in patients with severe *P*. *vivax* malaria was 21% (95% CI: 14–28%, I^2^: 86.8%). The prevalence reported in individual studies varied highly (range: 2–67%). Data on profound thrombocytopenia (13 cases) in patients with severe *P*. *knowlesi* malaria (60 cases) were available from two studies [30,53]. The PP of profound thrombocytopenia in patients with severe *P*. *knowlesi* malaria (77 cases) was 17% (95% CI: 8–26%, I^2^: 98.8%). The prevalence reported in individual studies varied highly (range: 6–26%). Overall, the PP of profound thrombocytopenia (202 cases) in patients with severe *P*. *vivax* and severe *P*. *knowlesi* malaria (1014 cases) was 20% (95% CI: 14–27%, I^2^: 85.2%) (Figure 3).

### 3.5. Difference in Platelet Counts between Patients with Severe and Uncomplicated Non-P. falciparum Malaria

**Figure 4:**
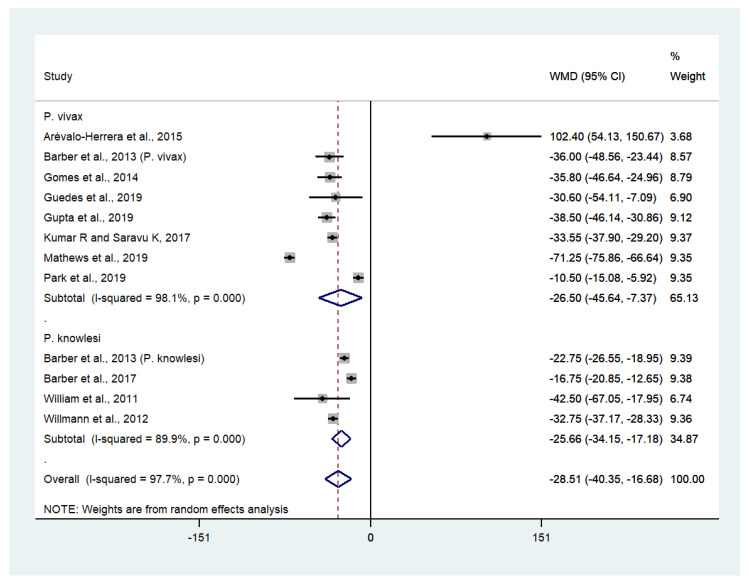
The difference in platelet counts between patients with severe and uncomplicated non-*P*. *falciparum* malaria. Abbreviations: WMD, weight mean difference; CI, confidence interval; black diamond symbol, point estimate; solid line in the middle of the graph at 0, zero effect size; dashed line: pooled WMD between the two groups; I^2^, level of heterogeneity; *p* = 0.00 or less than 0.05, significant heterogeneity.

Data on mean platelet counts in patients with severe *P*. *vivax* malaria (557 cases) and uncomplicated *P*. *vivax* malaria (2451 cases) were available from eight studies [27,30,33,34,35,38,43,45]. The pooled weighted mean difference (WMD) showed that the platelet counts of patients with severe *P*. *vivax* malaria were significantly lower than those of patients with uncomplicated *P*. *vivax* malaria (WMD: −26,500, 95% CI: −45,640 to −7370, Cochran’s Q < 0.0001, I^2^: 98.1%). Data on mean platelet counts in patients with severe *P*. *knowlesi* malaria (124 cases) and uncomplicated *P*. *knowlesi* malaria (318 cases) were available from four studies [29,30,53,54]. The pooled WMD showed that the platelet counts of patients with severe *P*. *knowlesi* malaria were significantly lower than those of patients with uncomplicated *P*. *knowlesi* malaria (WMD: −25,660, 95% CI: −34,150 to −17,180, Cochran’s Q < 0.0001, I^2^: 89.9%). Overall, the pooled WMD showed the platelet counts of patients with severe non-*P*. *falciparum* malaria (681 cases) were significantly lower than those of patients with uncomplicated non-*P*. *falciparum* malaria (2769 cases) (*p* < 0.001, WMD: −28,510, 95% CI: −40,350 to −16,680, Cochran’s Q < 0.0001, I^2^: 97.7%) (Figure 4).

### 3.6. Differences in Platelet Counts between Patients with Severe Non-P. falciparum and Severe P. falciparum Malaria

**Figure 5:**
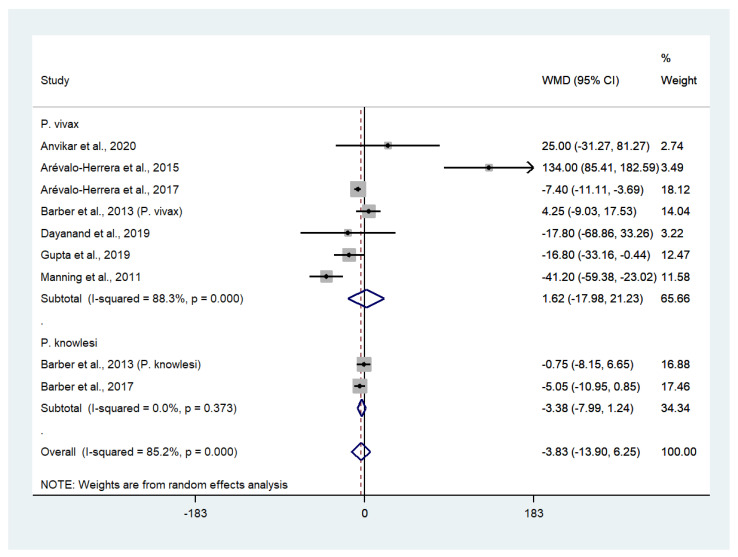
The absence of difference in platelet counts between patients with severe non-*P*. *falciparum* and severe *P*. *falciparum* malaria. Abbreviations: WMD, weight mean difference; CI, confidence interval; black diamond symbol, point estimate; solid line in the middle of the graph at 0, zero effect size; dashed line: pooled WMD between the two groups; I^2^, level of heterogeneity; *p* = 0.00 or less than 0.05, significant heterogeneity.

Data on mean platelet counts in patients with severe *P*. *vivax* malaria (252 cases) and severe *P*. *falciparum* malaria (576 cases) were available from seven studies [25,27,28,30,31,35,42]. The pooled WMD showed that the platelet counts in patients with severe *P*. *vivax* malaria and severe *P*. *falciparum* malaria were comparable (WMD: 16,300, 95% CI: −17,980 to 21,230, Cochran’s Q < 0.001, I^2^: 88.3%). Data on mean platelet counts in patients with severe *P*. *knowlesi* malaria (85 cases) and severe *P*. *falciparum* malaria (26 cases) were available from two studies [29,30]. The pooled WMD showed that the platelet counts of patients with severe *P*. *knowlesi* and severe *P*. *falciparum* malaria were comparable (WMD: 3380, 95% CI: −7990 to 1240, Cochran’s Q: 0.373, I^2^: 0%). Overall, the pooled WMD showed that the platelet counts of patients with severe non-*P*. *falciparum* (337 cases) and severe *P*. *falciparum* malaria (610 cases) were comparable (WMD: −3830, 95% CI: −1390 to 6250, Cochran’s Q < 0.0001, I^2^: 85.2%) (Figure 5).

### 3.7. Meta-Regression Analysis

A meta-regression analysis of the difference in platelet counts between patients with severe and uncomplicated non-*P*. *falciparum* malaria was performed using age, male ratio, and parasite counts as co-variates. The results of the univariate meta-regression analysis showed that the difference in platelet counts between the two groups was not confounded by age, male ratio, or parasite counts (*p* > 0.05, Figures S1–S3). A meta-regression analysis of the difference in platelet counts between patients with severe non-*P*. *falciparum* and severe *P*. *falciparum* malaria was also performed using age, male ratio, and parasite counts as co-variates. The results of the univariate meta-regression analysis showed that the difference in platelet counts between the two groups was not confounded by age, male ratio, or parasite counts (*p* > 0.05, Figures S4–S6).

### 3.8. Risk of Severe or Profound Thrombocytopenia in Patients with Severe Non-P. falciparum Malaria Compared with Uncomplicated Non-P. falciparum Malaria

**Figure 6:**
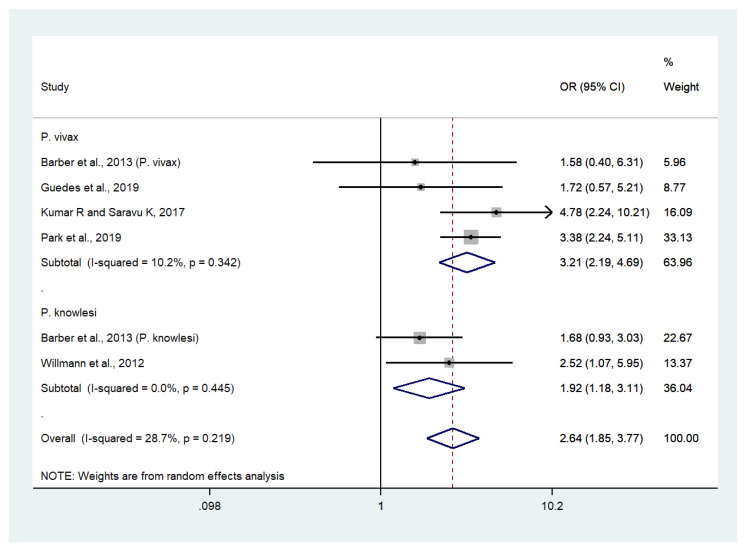
Risk of severe or profound thrombocytopenia in patients with severe non-*P*. *falciparum* malaria compared with uncomplicated non-*P*. *falciparum* malaria. Abbreviations: OR, odds ratio; CI, confidence interval; black diamond symbol, point estimate; solid line in the middle of the graph at 1, non-difference of effect size; dashed line: pooled OR between the two groups; I^2^, level of heterogeneity; *p* = 0.00 or less than 0.05, significant heterogeneity.

Data on severe or profound thrombocytopenia in patients with severe *P*. *vivax* malaria (403 cases) and uncomplicated *P*. *vivax* malaria (1647 cases) were available from five studies [30,34,38,45,54]. Patients with severe *P*. *vivax* malaria had significantly higher odds of severe or profound thrombocytopenia than those with uncomplicated *P*. *vivax* malaria (OR: 3.21, 95% CI: 2.19–4.69, Cochran’s Q: 0.34, I^2^: 10.2%). Data on severe thrombocytopenia in patients with severe *P*. *knowlesi* malaria (55 cases) and uncomplicated *P*. *knowlesi* malaria (185 cases) were available from two studies [30,54]. Patients with severe *P*. *knowlesi* malaria had significantly higher odds of severe or profound thrombocytopenia than those with uncomplicated *P*. *knowlesi* malaria (OR: 1.92, 95% CI: 1.18–3.11, Cochran’s Q: 0.445, I^2^: 0%). Overall, the odds of severe or profound thrombocytopenia were significantly higher in patients with severe non-*P*. *falciparum* malaria compared with those with uncomplicated non-*P*. *falciparum* malaria (OR: 2.64, 95% CI: 1.85–3.77, Cochran’s Q: 0.219, I^2^: 28.7%) (Figure 6).

### 3.9. Risk of Severe or Profound Thrombocytopenia in Patients with Severe Non-P. falciparum Malaria Compared with Severe P. falciparum Malaria

**Figure 7:**
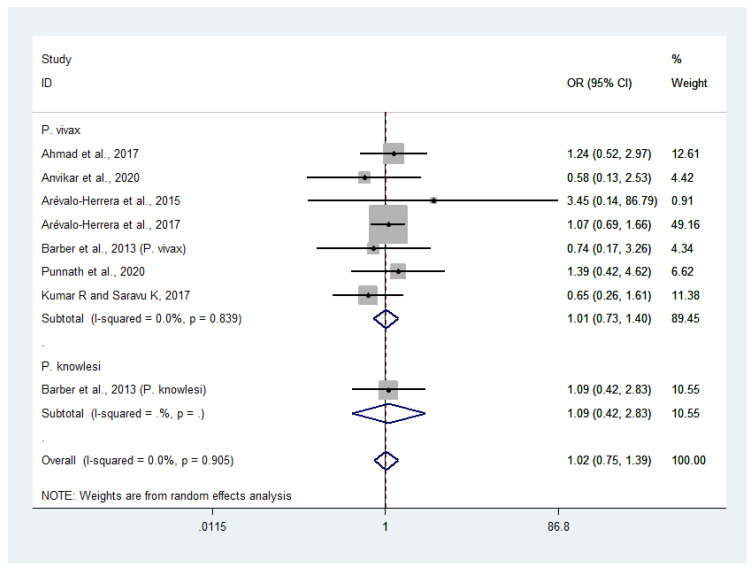
Risk of severe or profound thrombocytopenia in patients with severe non-*P*. *falciparum* malaria compared with severe *P*. *falciparum* malaria. Abbreviations: OR, odds ratio; CI, confidence interval; black diamond symbol, point estimate; solid line in the middle of the graph at 1, non-difference of effect size; dashed line: pooled OR between the two groups; I^2^, level of heterogeneity; *p* = 0.00 or less than 0.05, significant heterogeneity.

Data on severe or profound thrombocytopenia in patients with severe *P*. *vivax* malaria (381 cases) and severe *P*. *falciparum* malaria (396 cases) were available from seven studies [24,25,27,28,30,38,46]. The odds of severe or profound thrombocytopenia in patients with severe *P*. *vivax* and severe *P*. *falciparum* malaria were comparable (OR: 1.01, 95% CI: 0.73–1.40, Cochran’s Q: 0.839, I^2^: 0%). Data on severe thrombocytopenia in patients with severe *P*. *knowlesi* malaria (32 cases) and severe *P*. *falciparum* malaria (10 cases) were available from one study [30]. The results showed that the odds of severe or profound thrombocytopenia were comparable in patients with severe *P*. *knowlesi* and severe *P*. *falciparum* malaria (OR: 1.09, 95% CI: 0.42–2.83). Overall, no significant difference was observed in the odds of severe or profound thrombocytopenia between patients with severe non-*P*. *falciparum* and severe *P*. *falciparum* malaria (OR: 1.02, 95% CI: 75–1.39, Cochran’s Q: 0.905, I^2^: 0%) (Figure 7).

### 3.10. Deaths Related to Severe or Profound Thrombocytopenia

Data on deaths from severe *P*. *vivax* malaria were available for extraction. Deaths resulting from severe *P*. *vivax* malaria in patients with severe or profound thrombocytopenia were investigated. Mortality related to severe thrombocytopenia in patients with severe *P*. *vivax* malaria (63 cases with deaths, in three studies [28,39,50]) was 4% (95% CI: −2% to 10%), while mortality related to profound thrombocytopenia in patients with severe *P*. *vivax* malaria (21 cases with deaths) was 24% (95% CI: 0–45%) [49].

### 3.11. Publication Bias

**Figure 8:**
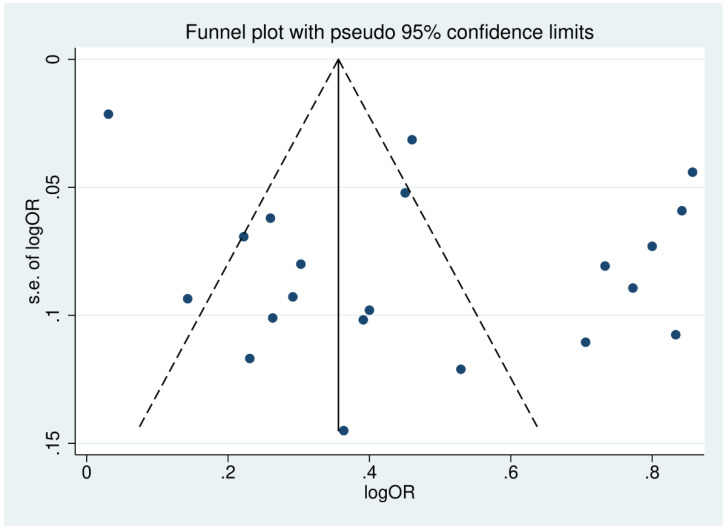
Funnel plot of included studies. The funnel plots showed the asymmetrical distribution of the logOR and the standard error (se) of logOR among studies reporting outcomes of patients with severe thrombocytopenia.

**Figure 9:**
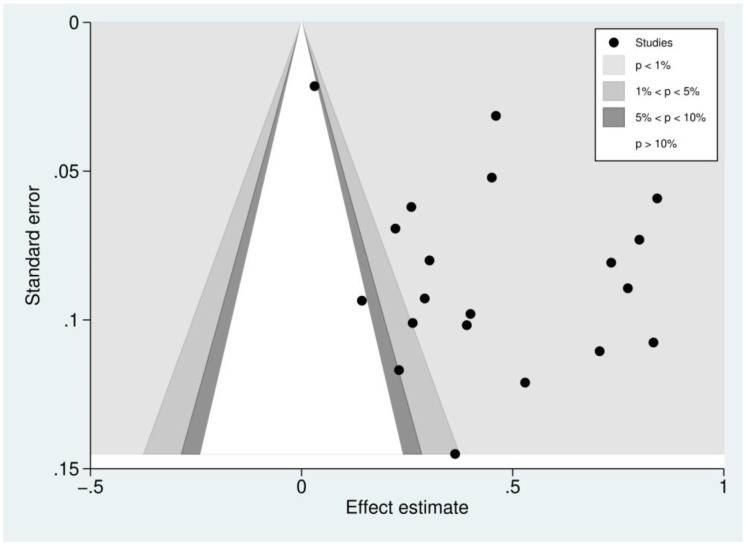
Contour-enhanced funnel plot of included studies. Funnel plot of included studies. The plots showed the distribution of the effect estimate (logOR) and the standard error (se) of logOR among studies reporting outcomes of patients with severe thrombocytopenia. Most of the effect estimates (black dots) located in the significant area (*p* < 0.01, 1%) indicated that the asymmetry of the funnel plot resulted from publication bias.

Publication bias was assessed by inspecting funnel plot asymmetry. Asymmetry was confirmed using an Egger’s test. The funnel plot of studies reporting the outcomes of patients with severe thrombocytopenia was asymmetrical, reflecting small-study effects (Figure 8). This asymmetry was confirmed by the Egger’s test, demonstrating the existence of small-study effects across the 21 included studies (*p* = 0.041, *t*-statistic: 2020, coefficient: 4.48, standard error: 2.04). A contour-enhanced funnel plot was generated and showed that the asymmetry of the funnel plot resulted from publication bias in most studies (Figure 9).

## 4. Discussion

Thrombocytopenia frequently occurs in individuals with *P*. *falciparum* and *P*. *vivax* malaria [55,56]. The greatest risks of severe thrombocytopenia were observed in individuals with *P*. *falciparum* single or mixed infections, accounting for >40% of cases [57]. In addition, the risk of severe thrombocytopenia was higher in patients with *P*. *falciparum* malaria than those with *P*. *vivax* malaria [57]. Moreover, severe malaria and mortality were more frequent in patients with *P*. *falciparum* malaria and severe thrombocytopenia [57,58,59]. To the best of our knowledge, this is the first systematic review and meta-analysis to examine severe and profound thrombocytopenia among individuals with severe non-*P*. *falciparum* malaria. Our results illustrate the high prevalence of severe thrombocytopenia in patients with severe *P*. *vivax* malaria (42%) and severe *P*. *knowlesi* malaria (80%). In addition to severe thrombocytopenia, we found that the PP of profound thrombocytopenia was low in patients with severe *P*. *vivax* malaria (21%) and severe *P*. *knowlesi* malaria (17%). The PP of severe and profound thrombocytopenia in patients with severe *P*. *vivax* malaria could not be precisely estimated because of the high heterogeneity among the included studies (I^2^ > 86%). In addition, the PP of severe thrombocytopenia in patients with severe *P*. *knowlesi* malaria could be reliably estimated because of the homogeneity of the included studies (I^2^: 0%). Nevertheless, we caution that the homogeneity among studies reporting the outcomes of patients with severe thrombocytopenia in *P*. *knowlesi* malaria could result from the relatively small number of included studies. Severe thrombocytopenia is not currently considered an indicator of severe malaria according to the WHO criteria [60]. Interestingly, in light of our analysis of severe and profound thrombocytopenia in patients with severe non-*P*. *falciparum* malaria, it may be worth investigating individuals with severe malaria and severe or profound thrombocytopenia. Clinicians managing patients with malaria should be aware of the risks, as these individuals are more likely to die than patients with higher platelet counts [14]. Furthermore, a previous study suggested that profound thrombocytopenia should be used as a severity criterion because it is a risk factor for mortality [57].

Thrombocytopenia is a common feature of malaria resulting from *P*. *falciparum*, *P*. *vivax*, and *P*. *knowlesi* infection [29,30,55]. The degree of thrombocytopenia relates to the high parasitemia of *Plasmodium* species [61]. Although thrombocytopenia is common in *Plasmodium* infections, significant bleeding is often unrelated to the level of thrombocytopenia [27]. Therefore, arguments have been made for routine platelet transfusions in malaria patients [62] and not defining thrombocytopenia as a clinical manifestation of severe malaria [60]. The potential diagnostic utility of severe or profound thrombocytopenia in individual patients is for the rapid treatment of severe malaria, as these patients may require additional support for clinical management [63]. The present study showed that the mean platelet counts of patients with severe *P*. *vivax* and severe *P*. *knowlesi* malaria were lower than those of patients with uncomplicated *P*. *vivax* and *P*. *knowlesi* malaria. Although high heterogeneity was present in the pooled analysis, these results demonstrated the significant diagnostic value of lower platelet counts in patients with severe non-*P*. *falciparum* malaria compared with patients with uncomplicated malaria. In the two-by-two meta-analysis of proportion, individuals with severe *P*. *vivax* malaria had more than three times the odds of developing severe or profound thrombocytopenia than those with uncomplicated *P*. *vivax* malaria. Lower odds of severe or profound thrombocytopenia were observed in patients with severe *P*. *knowlesi* malaria. The pooled ORs from the meta-analysis were reliable because of the low heterogeneity among the included studies. From the results of meta-analysis, most of the included studies showed lower mean platelet counts in patients with severe *P*. *vivax* malaria than in those with uncomplicated malaria [30,33,34,35,38,43,45], while only Arévalo-Herrera et al., 2015 [27] demonstrated higher mean platelet counts in patients with severe *P*. *vivax* malaria. Arévalo-Herrera et al., 2015 reported a wider range of platelet counts in patients with severe *P*. *vivax* malaria (82,000–660,000 cells/µL) than in those with uncomplicated *P*. *vivax* malaria, but no difference in the risk of spontaneous bleeding associated with thrombocytopenia [27]. Trends in mean platelet counts in patients with severe *P*. *knowlesi* malaria were similar to those in patients with severe *P*. *vivax* malaria: all included studies reported a significantly lower mean platelet count in these patients than in those with uncomplicated *P*. *knowlesi* malaria. Thrombocytopenia in patients with *P*. *knowlesi* infections is common, and platelet counts were lowest in cases of severe disease [53]. However, patients with severe *P*. *knowlesi* malaria exhibited higher mean platelet counts than those with severe *P*. *vivax* malaria.

We found no significant differences in the mean platelet counts of patients with severe *P*. *vivax/P*. *knowlesi* malaria and severe *P*. *falciparum* malaria. The two-by-two meta-analysis of proportion confirmed that neither severe nor profound thrombocytopenia was significantly different in patients with severe *P*. *vivax/P*. *knowlesi* malaria and severe *P*. *falciparum* malaria. The meta-analysis results were reliable because of the low heterogeneity in the pooled analysis of proportion. Our results demonstrated that severe and profound thrombocytopenia might affect the risk of death in individuals with severe *P*. *vivax* malaria. The data reported by four included studies [28,39,49,50] demonstrated that 84 individuals with severe *P*. *vivax* malaria exhibited severe and profound thrombocytopenia. Infection by other *Plasmodium* species, including *P*. *malariae* and *P*. *ovale* malaria, resulted in severe thrombocytopenia less frequently than *P*. *vivax* malaria [64,65] and *P*. *falciparum* malaria [65]. A previous study found that severe thrombocytopenia was significantly more common in patients with *P*. *ovale wallikeri* infection compared with those with *P*. *ovale curtisi* infection [66]. In *P*. *falciparum* infection, patients with profound thrombocytopenia were five times more likely to die than patients with higher platelet counts, suggesting that thrombocytopenia might be a marker of disease severity in patients with falciparum malaria [14,57]. The previous study that enrolled patients with *P*. *falciparum* and *P*. *vivax* suggested that severe thrombocytopenia had a sensitivity and specificity of 65.6% and 70.6%, respectively, to discriminate against severe malaria. Therefore, it is valuable to further investigate the sensitivity and specificity of thrombocytopenia using large longitudinal studies to conclude the performance of using platelet as an indicator for severe non-*P*. *falciparum* malaria. In light of our analyses for non-*P*. *falciparum* malaria, the mortality rate in patients with profound thrombocytopenia was higher than those with severe thrombocytopenia, suggesting the possibility of using thrombocytopenia to identify the risk of death in patients with severe non-*P*. *falciparum* malaria.

The pathogenic mechanisms underlying thrombocytopenia and the mechanisms through which platelet numbers affect severe malaria and death remain unclear. However, previous studies have shown that endothelial activation [67] results in platelet aggregation and clearance from circulation by the von Willebrand Factor (vWF) [68]. The role of platelet aggregation was supported by the finding that individuals with severe *P*. *falciparum* malaria deficient in a disintegrin and metalloprotease with thrombospondin type I repeats, member 13 (ADAMTS13), had higher frequencies of severe thrombocytopenia and other complications [69]. The high platelet consumption and activation observed in severe and fatal malaria cases may contribute to severe thrombocytopenia as platelet-expressed Toll-like receptors bind *P*. *falciparum* molecules and release prepackaged inflammatory mediators, leading to decreased signaling and inflammatory responses [70,71]. Another potential mechanism of severe thrombocytopenia-related malaria severity involves nitric oxide (NO). NO is a mediator of platelet homeostasis, and previous studies found a reduction in NO bioavailability in individuals with severe and fatal malaria [72].

In addition to identifying new candidate makers for severe malaria, new diagnostic tools for detecting malaria parasites are challenging. Recently, magnetic resonance relaxometry (MRR) [73,74,75], on-chip nuclear magnetic resonance (NMR) [76], rotating-crystal magneto-optical detection (RMOD) [77], and fluorescent blue-ray optical devices [78] have emerged as high-sensitivity malaria diagnostic tools to detect low parasite density in asymptomatic individuals. These new diagnostic tools will help increase the detection of malaria parasites and provide early treatment to patients, which can reduce malaria transmission, reducing the rate of misdiagnosis or mis-treatment that can facilitate severe malaria or anti-malarial drug resistance.

Biological and observational studies of non-*P*. *falciparum* malaria, particularly of *P*. *knowlesi* are fragmented, and is a research gap for malaria studies. The present study, Section 3.6 could only be derived from seven *P*. *vivax* studies and two *P*. *knowlesi* studies. Section 3.9 could only be derived from seven *P*. *vivax* studies and one *P*. *knowlesi* study, while Section 3.10 could only be derived from three *P*. *vivax* studies. It would be interesting to determine whether such comparability in the severity or profoundness of thrombocytopenia and related outcomes was due to the limitations in the available studies or unique biological traits that exist within non-*P*. *falciparum* species that induce host responses. The detection of human cases of *P*. *knowlesi* demonstrated an ongoing increase despite effective control programs to disrupt the transmission of human-only *Plasmodium* species [77]. Some notable concern in terms of a potential increase in human infections with *P*. *knowlesi* malaria can be found in the changing patterns in land use where spillover opportunities for human infection due to increased contact with natural reservoirs and infected vectors have been aggravated. In addition, the range and distribution of primary hosts and vectors including their bionomics are still lacking in data [78]. These are strong rationales to conduct further studies on the parasite, vector, and host biology in non-*P*. *falciparum* malaria and host immunological responses to the same.

Our study had several limitations. First, the data on platelet counts and the frequency of severe or profound thrombocytopenia could only be extracted from studies of patients with severe *P*. *vivax* and severe *P*. *knowlesi* malaria. Therefore, the prevalence of severe or profound thrombocytopenia was not assessed among patients with severe *P*. *malariae*, *P*. *ovale*, or mixed malaria. Second, the prevalence of severe or profound thrombocytopenia-related death could not be precisely estimated because of the limited number of included studies. Despite these limitations, the presence of severe or profound thrombocytopenia in malaria patients may support the diagnosis of malaria, particularly in patients with severe non-*P*. *falciparum* malaria, because low levels of parasitemia are frequently encountered in routine diagnosis. In addition, severe or profound thrombocytopenia could be used as an indicator of increased risk of poor outcome and mortality if there are delays in diagnosis. Our results suggest that severe and/or profound thrombocytopenia should be included in the WHO criteria for severe non-*P*. *falciparum* malaria because a high prevalence of severe thrombocytopenia was observed in these patients (47% for severe thrombocytopenia and 20% for profound thrombocytopenia).

The present systematic review provided the new insight into the clinical relevance of severe and profound thrombocytopenia in diagnosing and managing non-*P*. *falciparum* malaria. Severe thrombocytopenia should serve as a warning sign of increased chances of complications. Meanwhile, profound thrombocytopenia should serve as a marker of mortality among patients with non-*P*. *falciparum* malaria.

## 5. Conclusions

A meta-analysis provides enhanced statistical power, thereby providing more robust and reliable knowledge of the effects of severe non-*P*. *falciparum* malaria and thrombocytopenia. Our results suggest that individuals with severe non-*P*. *falciparum* malaria, particularly *P*. *vivax* and *P*. *knowlesi* malaria, with severe or profound thrombocytopenia should be regarded as high-risk patients and should subsequently be treated for severe malaria according to the current WHO guidelines. In addition, severe or profound thrombocytopenia coupled with other clinical and microscopy parameters, may significantly improve malaria diagnosis, provide timely treatment for malaria infections, and reduce the morbidity and mortality of severe non-*P*. *falciparum* malaria. Observational studies and research on the parasite, vector, and host biology and outcomes for non-*P*. *falciparum* malaria is still fragmented. Moreover, the changing patterns of land use that increase the exposure of natural reservoirs and infected vectors, range distribution of hosts, and vectors, including the biomics of non-*P*. *falciparum* malaria, require further and more in-depth exploration.

## Data Availability

All data relating to the present study are available in this manuscript.

## Supplementary Materials

The following are available online at https://www.mdpi.com/article/10.3390/biology10121275/s1, Figure S1. Results of univariate meta-regression analysis showing differences in platelet counts between patients with severe and uncomplicated non-*P*. *falciparum* malaria using age as a covariate; Figure S2. Results of univariate meta-regression analysis showing differences in platelet counts between patients with severe and uncomplicated non-*P*. *falciparum* malaria using the male ratio as a covariate; Figure S3. Results of univariate meta-regression analysis showing differences in platelet counts between patients with severe and uncomplicated non-*P*. *falciparum* malaria using parasite count ratio as a covariate; Figure S4. Results of univariate meta-regression analysis showing differences in platelet counts between patients with severe non-*P*. *falciparum* and severe *P*. *falciparum* malaria using age as a covariate; Figure S5. Results of univariate meta-regression analysis showing differences in platelet counts between patients with severe non-*P*. *falciparum* and severe *P*. *falciparum* malaria using the male ratio as a covariate; Figure S6. Results of univariate meta-regression analysis showing differences in platelet counts between patients with severe non-*P*. *falciparum* and severe *P*. *falciparum* malaria using parasite count ratio as a covariate; Table S1. Characteristics of the included studies; Table S2. Risk of bias in the included studies. PRISMA Checklist S1. Guidelines for reporting systematic review and meta-analysis.
