# Supplementary material for: Alteration of Platelet Count in Patients with Severe Non-Plasmodium falciparum Malaria: A Systematic Review and Meta-Analysis
Source: Biology (Basel). 2021 Dec 5;10(12):1275. doi: 10.3390/biology10121275 (PMC8698983; doi:10.3390/biology10121275)
Supplement: Supplementary file 1 [file biology-10-01275-s001.zip › biology-1457993-supplementary.pdf]

Supplementary Materials

Alteration of Platelet Count in Patients with Severe Non-*Plasmodium falciparum* Malaria: A Systematic Review and Meta-Analysis

Aongart Mahittikorn, Frederick Ramirez Masangkay, Kwuntida Uthaisar Kotepui, Wanida Mala, Giovanni De Jesus Milanez, Polrat Wilairatana and Manas Kotepui

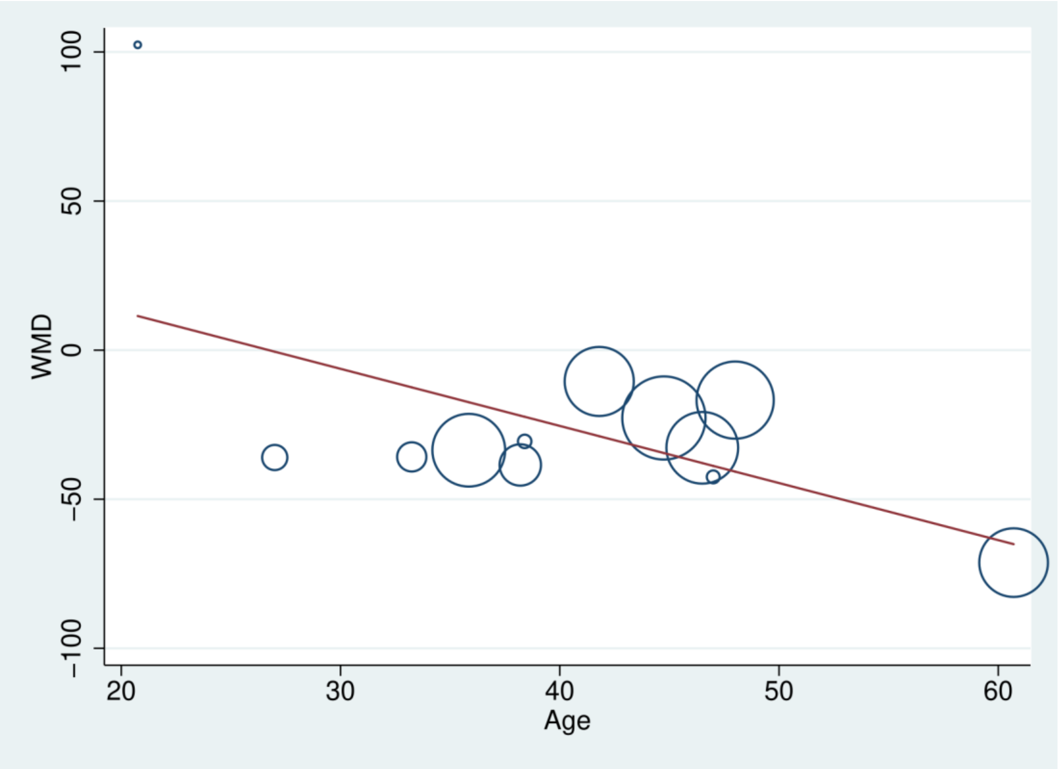

Figure S1. Results of the univariate meta-regression analysis showing the difference in platelet counts between severe and uncomplicated non-*P. falciparum* malaria using age as a covariate.

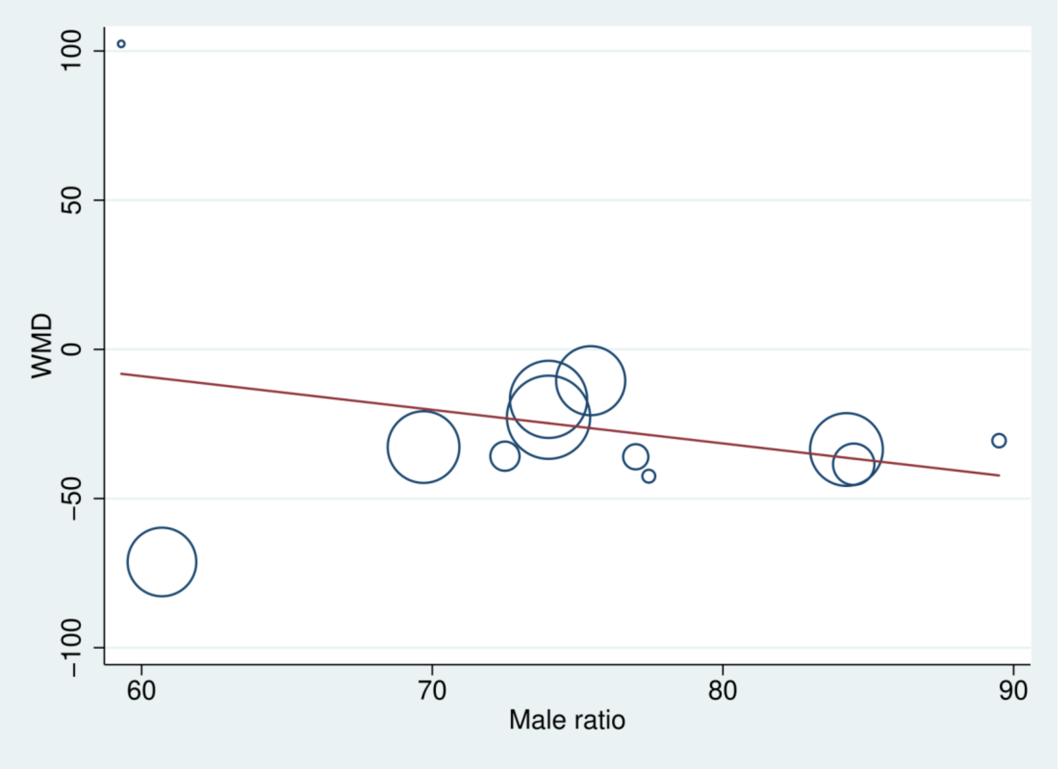

Figure S2. Results of the univariate meta-regression analysis showing the difference in platelet counts between severe and uncomplicated non-P. falciparum malaria using the male ratio as a covariate.

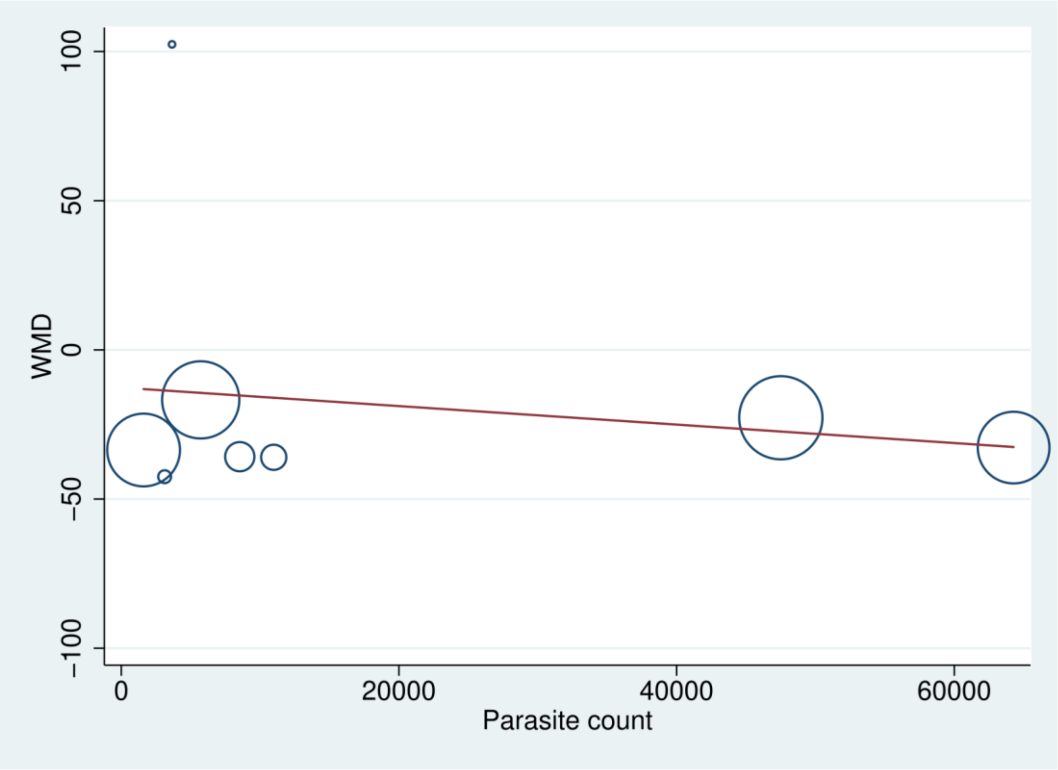

Figure S3. Results of the univariate meta-regression analysis showing the difference in platelet counts between severe and uncomplicated non-*P. falciparum* malaria using the parasite count ratio as a covariate.

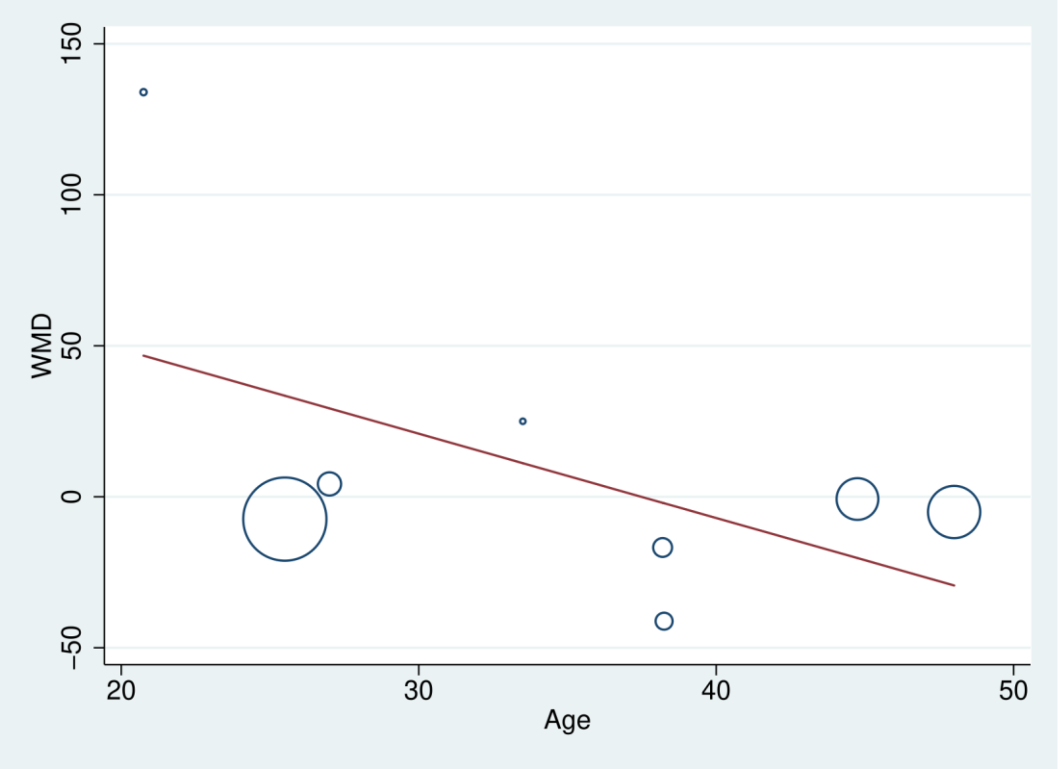

Figure S4. Results of the univariate meta-regression analysis showing the difference in platelet counts between severe non-*P. falciparum* and severe *P. falciparum* malaria using age as a covariate.

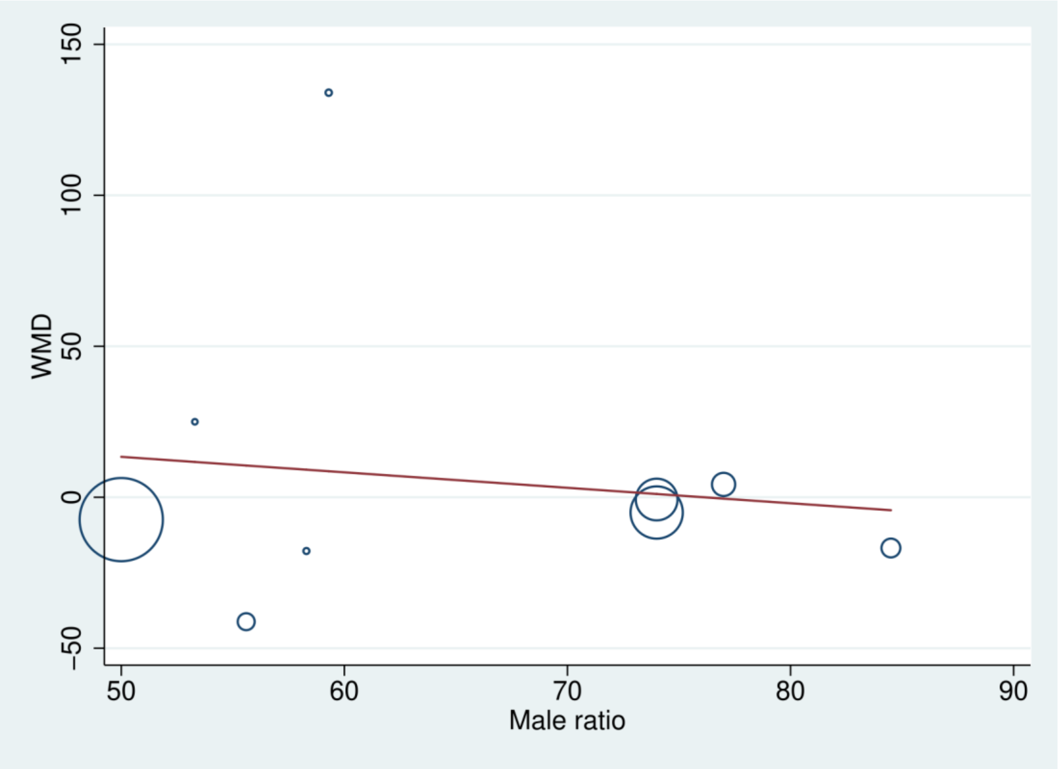

Figure S5. Results of the univariate meta-regression analysis showing the difference in platelet counts between severe non-*P. falciparum* and severe *P. falciparum* malaria using the male ratio as a covariate.

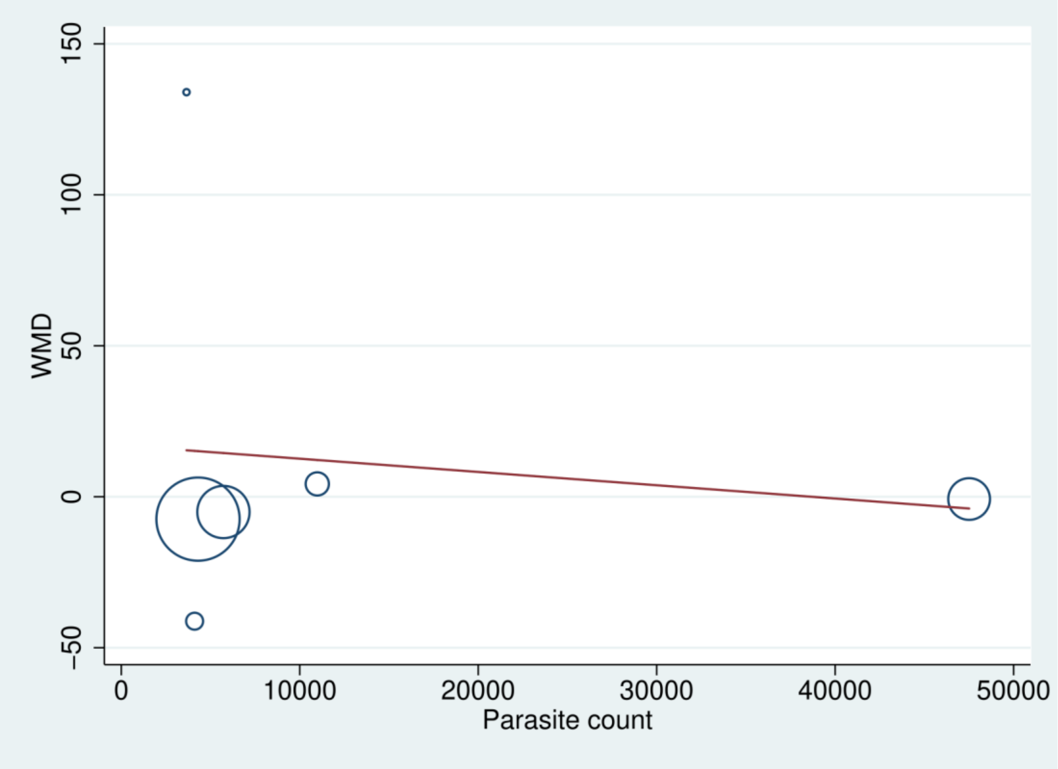

Figure S6. Results of the univariate meta-regression analysis showing the difference in platelet counts between severe non-*P. falciparum* and severe *P. falciparum* malaria using the parasite count ratio as a covariate.

Table S1. Characteristics of the included studies.

Databases	Search terms	Date
MEDLINE	(Severe OR complicated OR complication) AND (malaria OR plasmodium) AND (thrombocytopenia OR "low platelet")	1 June 2020 to 9 June 2020
Scopus	(Severe OR complicated OR complication) AND (malaria OR plasmodium) AND (thrombocytopenia OR "low platelet")<br>Search option: All fields	1 June 2020 to 9 June 2020
ISI Web of Science	(Severe OR complicated OR complication) AND (malaria OR plasmodium) AND (thrombocytopenia OR "low platelet")<br>Search option: All fields	1 June 2020 to 9 June 2020

Table S2. Risk of bias in the included studies.

		**Selection**					**Exposure**				
**No.**	**Reference**	**Is the Case Definition Adequate?**	**Representativeness of the Cases**	**Selection of Controls**	**Definition of Controls**	**Compatibility**	**Ascertainment of Exposure**	**Same method of ascertainment for cases and controls**	**Non – Response Rate**	**Total score (3)**	**Rating (High, moderate, low quality)**
**1.**	**Ahmad et al., 2017**			NA	NA	NA		NA	NA	3	High
2	Alexandre et al., 2010			NA	NA	NA		NA	NA	3	High
3.	Anvikar et al., 2020			NA	NA	NA		NA	NA	3	High
4.	Arboleda et al., 2012			NA	NA	NA		NA	NA	3	High
5.	Arévalo-Herrera et al., 2015			NA	NA	NA		NA	NA	3	High
6.	Arévalo-Herrera et al., 2017			NA	NA	NA		NA	NA	3	High
7.	Barber et al., 2013			NA	NA	NA		NA	NA	3	High
8.	Barber et al., 2017			NA	NA	NA		NA	NA	3	High
9.	Dayanand et al., 2019			NA	NA	NA		NA	NA	2	High
10.	Douglas et al., 2014			NA	NA	NA		NA	NA	3	High
11.	Gomes et al., 2014			NA	NA	NA		NA	NA	2	Moderate
12.	Guedes et al., 2019			NA	NA	NA		NA	NA	3	High
13.	Gupta et al., 2016			NA	NA	NA		NA	NA	3	High
14.	Gupta et al., 2019			NA	NA	NA		NA	NA	3	High
15.	Jain et al., 2013			NA	NA	NA		NA	NA	3	High
16.	Kumar R and Saravu K, 2017			NA	NA	NA		NA	NA	3	High
17.	Kumari M and Ghildiyal R, 2014			NA	NA	NA		NA	NA	3	High
18.	Kute et al., 2012			NA	NA	NA		NA	NA	3	High
19.	Lanca et al., 2012			NA	NA	NA		NA	NA	3	High
20.	Manning et al., 2011			NA	NA	NA		NA	NA	3	High
21.	Mathews et al., 2019			NA	NA	NA		NA	NA	3	High
22.	O'Brien et al., 2014			NA	NA	NA		NA	NA	3	High
23.	Park et al., 2019			NA	NA	NA		NA	NA	3	High
24.	Punnath et al., 2020			NA	NA	NA		NA	NA	3	High
25.	Rajeshwar K and Karibasappa BG, 2015			NA	NA	NA		NA	NA	3	High
26.	Raposo et al., 2013			NA	NA	NA		NA	NA	3	High
27.	Sharma et al., 2012			NA	NA	NA		NA	NA	2	Moderate
28.	Singh et al., 2011			NA	NA	NA		NA	NA	3	High
29.	Sud et al., 2012			NA	NA	NA		NA	NA	3	High
30.	Tanwar et al., 2012			NA	NA	NA		NA	NA	3	High
31.	Val et al., 2017			NA	NA	NA		NA	NA	3	High
32.	William et al., 2011			NA	NA	NA		NA	NA	2	Moderate
33.	Willmann et al., 2012			NA	NA	NA		NA	NA	2	Moderate

NA: Not assessed.

PRISMA 2009 Checklist

Section/topic	#	Checklist item	Reported on page #
**TITLE**			
Title	1	Identify the report as a systematic review, meta-analysis, or both.	Title, paragraph 1<br>Page 1
**ABSTRACT**			
Structured summary	2	Provide a structured summary including, as applicable: background; objectives; data sources; study eligibility criteria, participants, and interventions; study appraisal and synthesis methods; results; limitations; conclusions and implications of key findings; systematic review registration number.	Abstract, paragraph 1<br>Page 1-3
**INTRODUCTION**			
Rationale	3	Describe the rationale for the review in the context of what is already known.	Background, paragraph 1-2<br>Page 4-5
Objectives	4	Provide an explicit statement of questions being addressed with reference to participants, interventions, comparisons, outcomes, and study design (PICOS).	Background, paragraph 2<br>Page 5
**METHODS**			
Protocol and registration	5	Indicate if a review protocol exists, if and where it can be accessed (e.g., Web address), and, if available, provide registration information including registration number.	Methods, paragraph 1<br>Page 5
Eligibility criteria	6	Specify study characteristics (e.g., PICOS, length of follow-up) and report characteristics (e.g., years considered, language, publication status) used as criteria for eligibility, giving rationale.	Methods, paragraph 2<br>Page 5-6
Information sources	7	Describe all information sources (e.g., databases with dates of coverage, contact with study authors to identify additional studies) in the search and date last searched.	Methods, paragraph 3<br>Page 6-7

Search	8	Present full electronic search strategy for at least one database, including any limits used, such that it could be repeated.	Methods, paragraph 3 Page 6-7
Study selection	9	State the process for selecting studies (i.e., screening, eligibility, included in systematic review, and, if applicable, included in the meta-analysis).	Methods, paragraph 3 Page 6-7
Data collection process	10	Describe method of data extraction from reports (e.g., piloted forms, independently, in duplicate) and any processes for obtaining and confirming data from investigators.	Methods, paragraph 3 Page 6-7
Data items	11	List and define all variables for which data were sought (e.g., PICOS, funding sources) and any assumptions and simplifications made.	Methods, paragraph 3 Page 6-7
Risk of bias in individual studies	12	Describe methods used for assessing risk of bias of individual studies (including specification of whether this was done at the study or outcome level), and how this information is to be used in any data synthesis.	Methods, paragraph 4<br><br>Page 7
Summary measures	13	State the principal summary measures (e.g., risk ratio, difference in means).	Methods, paragraph 5 Page 7-8
Synthesis of results	14	Describe the methods of handling data and combining results of studies, if done, including measures of consistency (e.g., I^2) for each meta-analysis.	Methods, paragraph 5 Page 7-8

**Section/topic**	**#**	**Checklist item**	**Reported on page #**
Risk of bias across studies	15	Specify any assessment of risk of bias that may affect the cumulative evidence (e.g., publication bias, selective reporting within studies).	Methods, paragraph 5 Page 7-8
Additional analyses	16	Describe methods of additional analyses (e.g., sensitivity or subgroup analyses, meta-regression), if done, indicating which were pre-specified.	Methods, paragraph 5

			Page 7-8
**RESULTS**			
Study selection	17	Give numbers of studies screened, assessed for eligibility, and included in the review, with reasons for exclusions at each stage, ideally with a flow diagram.	Results, paragraph 1<br>Page 8
Study characteristics	18	For each study, present characteristics for which data were extracted (e.g., study size, PICOS, follow-up period) and provide the citations.	Results, paragraph 2<br>Page 8-9
Risk of bias within studies	19	Present data on risk of bias of each study and, if available, any outcome level assessment (see item 12).	Results, paragraph 3<br>Page 9
Results of individual studies	20	For all outcomes considered (benefits or harms), present, for each study: (a) simple summary data for each intervention group (b) effect estimates and confidence intervals, ideally with a forest plot.	Results, paragraph 4-10<br>Page 8-13
Synthesis of results	21	Present results of each meta-analysis done, including confidence intervals and measures of consistency.	Results, paragraph 4-10<br>Page 8-13
Risk of bias across studies	22	Present results of any assessment of risk of bias across studies (see Item 15).	Results, paragraph 15<br>Page 14
Additional analysis	23	Give results of additional analyses, if done (e.g., sensitivity or subgroup analyses, meta-regression [see Item 16]).	Results, paragraph 8<br>Page 11-12

**DISCUSSION**			
Summary of evidence	24	Summarize the main findings including the strength of evidence for each main outcome; consider their relevance to key groups (e.g., healthcare providers, users, and policy makers).	Discussion, paragraph 1-4<br>Page 14-17
Limitations	25	Discuss limitations at study and outcome level (e.g., risk of bias), and at review-level (e.g., incomplete retrieval of identified research, reporting bias).	Discussion, paragraph 5<br>Page 17-18
Conclusions	26	Provide a general interpretation of the results in the context of other evidence, and implications for future research.	Conclusion, paragraph 6<br>Page 18
**FUNDING**			
Funding	27	Describe sources of funding for the systematic review and other support (e.g., supply of data); role of funders for the systematic review.	Funding, paragraph 1<br>Page 13

From: Moher D, Liberati A, Tetzlaff J, Altman DG, The PRISMA Group (2009). Preferred Reporting Items for Systematic Reviews and Meta-Analyses: The PRISMA Statement. PLoS Med 6(7): e1000097. doi:10.1371/journal.pmed1000097

For more information, visit: **www.prisma-statement.org**.
